# Comparison of Lipid Content in Nine Dinoflagellate Species Using Flow Cytometry

**DOI:** 10.3390/microorganisms13010044

**Published:** 2024-12-30

**Authors:** Jaeyeon Park, Eun Young Yoon, Seung Joo Moon, Jun-Ho Hyung, Hangy Lee

**Affiliations:** Advanced Institute of Convergence Technology, Suwon 16229, Republic of Korea; sjmoon04@snu.ac.kr (S.J.M.); hjh1120@snu.ac.kr (J.-H.H.); leehg97@snu.ac.kr (H.L.)

**Keywords:** dinoflagellate, lipid content, flow cytometry, carbon content, cell size

## Abstract

The lipid content of nine dinoflagellates was analyzed using flow cytometry to compare lipid levels. Additionally, the correlation between lipid content, cell size, and carbon content in dinoflagellates was evaluated using BODIPY 505/515 staining. The flow cytometry side scatter (SSC) effectively represented relative cell size, showing a linear relationship with the equivalent spherical diameter (ESD). Larger cells exhibited higher relative lipid content; however, lipid accumulation was influenced by nutritional modes and habitats, with mixorophic and benthic species displaying higher lipid content than heterotrophic species. A comparison of fluorescent dyes revealed that Nile Red overestimated lipid content, suggesting overlap with chlorophyll autofluorescence. Flow cytometry analysis with BODIPY 505/515 demonstrated a linear correlation with the sulfo-phospho-vanillin (SPV) method, enabling determination of actual lipid content using FL1 fluorescence and the slope value. As the carbon content increased, the lipid content initially increased rapidly but plateaued at higher carbon levels, indicating saturation. These findings suggest that relative fluorescence via flow cytometry provides an effective means to estimate the lipid content and carbon content as a function of cell size.

## 1. Introduction

Marine dinoflagellates contain large amounts of high-quality lipids [1,2]. As major contributors to the marine food web, they play crucial ecological roles and are often associated with the formation of red tides [3,4]. Owing to their explosive and extensive blooms with high cell concentrations, they are considered a potential source of biofuel [5,6,7,8,9]. Thus, knowing the lipid content and lipid productivity of each species is very important when selecting target species or potential candidates as a source of biofuel. Some studies have been conducted for selecting the optimal species of dinoflagellate as a candidate for lipid production [10,11,12].

However, analyzing the lipid content of marine dinoflagellate species often requires clonal cultures and large amounts of biomass. Establishing clonal cultures for some dinoflagellates can be challenging, and mass cultivation of these species is time-consuming and labor-intensive. For instance, direct lipid extraction methods, such as those developed by Folch et al. [13] and Bligh and Dyer [14], which use chloroform and methanol, require substantial biomass. To address this limitation, several studies have explored methods for estimating lipid content in marine plankton species using smaller amounts of biomass. One example is the sulfo-phospho-vanillin method [15,16,17,18,19].

Flow cytometry is a rapid and effective method for quantitative analysis of individual cells in a moving fluid. Microalgae are ideal for flow cytometry analysis because they are unicellular and contain photosynthetic pigments that exhibit autofluorescence [20]. Moreover, the flow cytometric measurement of algal lipid content is one of the most cost-effective and time-efficient methods for labeling cells with lipophilic fluorescent dyes [21,22].

Traditionally, algal lipid content has been measured using flow cytometry in cells labeled with Nile Red [6,23,24]. However, Nile Red fluorescence emission often overlaps with chlorophyll autofluorescence, leading to spectral interference [22,25]. To address this limitation, BODIPY 505/515 (4,4-Difluoro-1,3,5,7-Tetramethyl-4-Bora-3a,4a-Diaza-s-Indacene) was investigated as an alternative fluorescent dye for algal lipid analysis [21,22,25,26]. BODIPY 505/515 has a narrower emission spectrum than Nile Red and its fluorescence emission is significantly offset by chlorophyll autofluorescence, thereby eliminating spectral interference [26,27].

In this study, we assessed the applicability of flow cytometry for accurate lipid estimation in dinoflagellates. We analyzed the lipid content of nine dinoflagellate species (*Amphidinium carterae*, *Kryptoperidinium triquetrum*, *Prorocentrum cordatum*, *Alexandrium minutum*, *Oxyrrhis marina*, *Scrippsiella acuminata*, *Ostreopsis ovata*, *Prorocentrum micans*, *Lingulaulax polyedra*) using flow cytometry with a fluorescent dye. Additionally, correlations were established between the estimated lipid content, cell size, lipid content measured using the sulfo-phospho-vanillin (SPV) method, and carbon content.

## 2. Materials and Methods

### 2.1. Preparation of Experimental Organisms

We prepared nine dinoflagellate species for comparison, including one benthic dinoflagellate and one heterotrophic dinoflagellate (Table 1). Cultures were grown at 20 °C in enriched f/2-Si seawater medium [28] under continuous illumination of 20 μE m^−2^ s^−1^ provided by cool white fluorescent lights, except for *O. marina*. Yeast was used to culture the heterotrophic dinoflagellate *O. marina* at 20 °C in autoclaved seawater.

Experimental organisms were cultured in 2 L polycarbonate bottles containing 1 L of f/2-Si medium. Each culture was grown for over one week until the end of the exponential growth phase to stationary phase, with cell concentrations exceeding 3000 cells mL^−1^. A 10 mL sample from each culture was fixed with Lugol’s solution for cell concentration counts, and another 10 mL sample was prepared for flow cytometric analysis.

The mean equivalent spherical diameter (ESD) ± standard deviation was measured using an electronic particle counter (Coulter Multisizer II; Coulter Corporation, Miami, FL, USA).

The biovolume (μm^3^) of each dinoflagellate was calculated using the method described by Hillebrand et al. [29]. The biovolume was calculated using the ESD and the equation for spherical-shaped cells.

$$BV=\frac{4}{3}\cdot \pi \cdot r^{3}$$

Once cell biovolume was calculated, carbon content per cell (pg cell^−1^) was assessed using conversion factors from the literature described by Menden-Deuer and Lessard [30] (pg C cell^−1^ = 0.216 × BV^0.939^). The calculated carbon content was compared to the lipid content estimated using flow cytometry.

### 2.2. Staining of Algal Cells Using Fluorescent Dyes

The algal cells were stained with BODIPY 505/515 (4,4-Difluoro-1,3,5,7-Tetramethyl-4-Bora-3a,4a-Diaza-s-Indacene; Sigma-Aldrich, St Louise, MO, USA) following a protocol adapted from Cooper et al. [14]. BODIPY 505/515 was dissolved in anhydrous dimethyl sulfoxide (DMSO) to prepare a 10 mM stock solution, which was stored in an amber bottle to protect it from light. For staining, 2 μL of the stock solution was added to 10 mL of live phytoplankton samples to achieve a final concentration of 2 μM. The samples were incubated for 10 min at room temperature.

For Nile Red staining, the cultured cells were stained using a modified protocol from Dempster and Sommerfeld [31]. A working solution of Nile Red (NR; Sigma-Aldrich, St Louise, MO, USA) was prepared by dissolving NR in acetone (0.1 mg mL^−1^). For staining, 50 μL of the working solution was added to 10 mL of the cultures, gently vortexed, and incubated in the dark at 37 °C for 10 min.

### 2.3. Flow Cytometric Analysis

The relative fluorescence of lipids was determined using a FACSCalibur flow cytometer (BD Biosciences, San Jose, CA, USA) equipped with a 488 nm argon laser. The fluorescence of cells labeled with BODIPY 505/515 was measured in the FL1 channel using a FITC filter for green light (530 ± 15 nm). For cells labeled with Nile Red, fluorescence was collected in the FL2 channel to capture yellow and orange light (560–640 nm). Approximately 3000 cells were analyzed for each sample, and fluorescence signals were recorded using logarithmic amplification. Non-stained cells were used as autofluorescence controls. To estimate cell size, side-scattered light (SSC) was used because the experimental cells were mostly spherical in shape.

The photomultiplier voltage was adjusted according to the size and fluorescence characteristics of the organism. Flow cytometry is typically employed to examine picoplankton and nanoplankton samples; it necessitated adjustments to the voltage settings to analyze marine dinoflagellates, which have dimensions ranging from 5 to 50 μm.

To ensure consistent detection across all experimental organisms, we mixed *A. carterae* (9.8 μm, smallest) and *L. polyedra* (38 μm, largest) and measured SSC and FL1 fluorescence using BODIPY 505/515 and FL2 fluorescence using Nile Red. The voltage was adjusted to accommodate the size range and maximum fluorescence of both the dyes. The final voltage gain settings used for analysis were Forward Scatter (FSC) = E00, Side Scatter (SSC) = 250, green fluorescence (FL1) = 220, and orange fluorescence (FL2) = 300.

**Figure 1:**
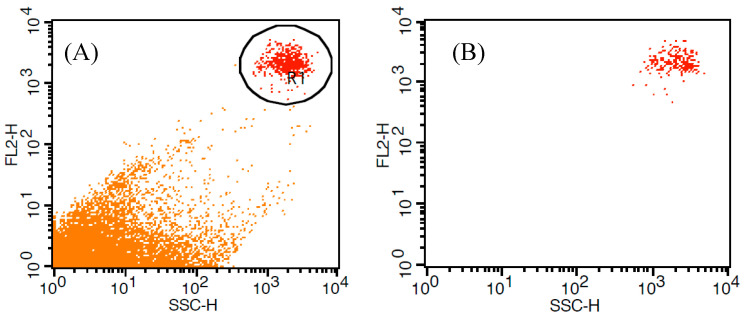
Gating of dinoflagellate *Prorocentrum micans* to exclude non-fluorescent particles, bacteria, and detritus. (**A**) *P. micans* labelled with Nile Red and fluorescence measurements using FL2. R1 was the selected gate that contained labelled *P. micans* only. (**B**) Measurement of the gated population (R1 only).

To isolate the experimental organism population, gates were established to exclude nonfluorescent particles. The gated algal populations typically constituted the dominant events detected (Figure 1). Measurements of the gated algal populations were performed in triplicate for each experimental species.

## 3. Results and Discussion

### 3.1. Flow Cytometric Analysis of Lipid Contents Using BODIPY 505/515

**Figure 2:**
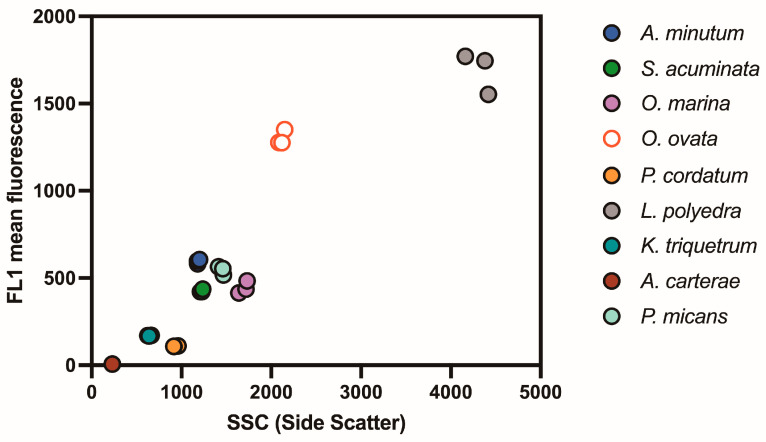
Flow cytometry analysis of lipid content in experimental dinoflagellate species with BODIPY 505/515 and Side Scatter (SSC) as size factors.

**Figure 3:**
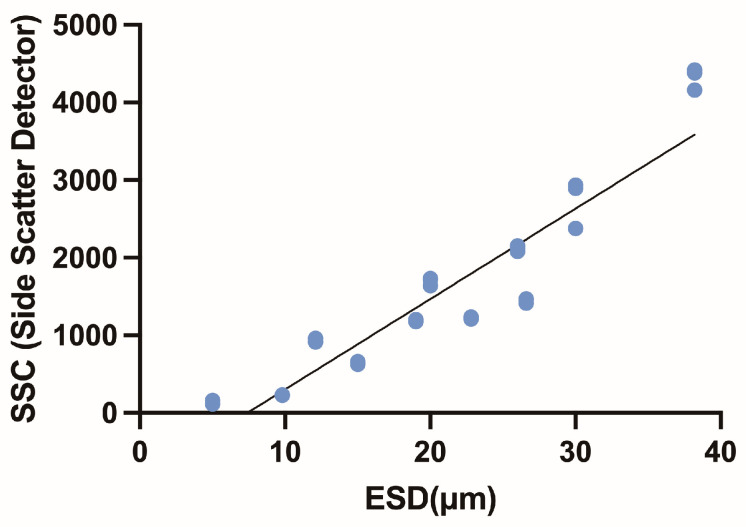
Correlation between experimental cell size measured with a Coulter counter (*X*-axis, ESD) and flow cytometry (SSC: Side Scatter Detector fluorescence). R^2^ value is 0.86.

The FL1 fluorescence of BODIPY 505/515-stained cells and side scatter (size factor) detected by flow cytometry are shown in Figure 2. The lipid content of the experimental dinoflagellate species, estimated by FL1 fluorescence, increased with the side scatter (size factor) measured by flow cytometry (Figure 2, Table 2). A linear relationship (R^2^ = 0.86, Figure 3) was observed between two size factors: side scatter (SSC) from flow cytometry and equivalent spherical diameter (ESD, μm) from a Coulter Multisizer. As most experimental species had spherical cell shapes, the SSC absorbance closely matched the ESD and effectively represented the relative cell size.

**Figure 4:**
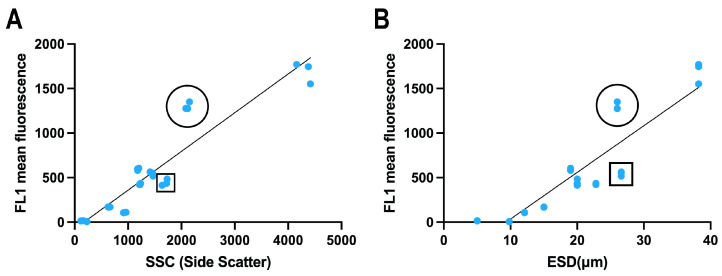
Correlation between the lipid content of nine dinoflagellate species labelled with BODIPY 505/515 and the size factor. (**A**) Size factor measured by flow cytometry (SSC); (**B**) size measured by Coulter Multisizer (ESD). Open circles indicate *Ostreopsis ovata* and open squares indicate *Prorocentrum micans*. R^2^ value is 0.87 and 0.81, respectively.

The correlation between relative lipid content (BODIPY 505/515 fluorescence) and two size factors was compared for the dinoflagellate species (Figure 4). Both size factors were significantly correlated with BODIPY 505/515 fluorescence, with larger cells exhibiting a higher relative lipid content. When the size was measured using flow cytometry (SSC), *O. ovata* displayed greater fluorescence than the other dinoflagellate species examined (Figure 4A).

Similarly, when the size was determined as the equivalent spherical diameter (ESD) using a Coulter Multisizer, *P. micans* and *O. ovata* exhibited comparable ESD sizes; however, *O. ovata* displayed significantly higher FL1 fluorescence (Figure 4B). The size of *P. micans* was underestimated when measured by flow cytometry, likely because of its distinctive cell shape, which is the form of teardrop with a wide valve and appears lenticular in the lateral view as a result of compression.

### 3.2. Comparision of Fluorescent Dyes (BODIPY 505/515 vs. Nile Red)

For decades, Nile Red has been widely used to stain lipid droplets in microalgal cells. Previous studies reported that diatoms and dinoflagellates are more suitable for fluorescent measurement due to the cell wall structure. Consequently, Nile Red and BODIPY 505/515 staining can be used as high-throughput approaches without the need for pre-treatment [32]. However, many studies have highlighted several issues associated with the use of Nile Red. Its major disadvantages include limited photostability, interference with chlorophyll fluorescence, and difficulty permeating certain microalgal species [33].

**Figure 5:**
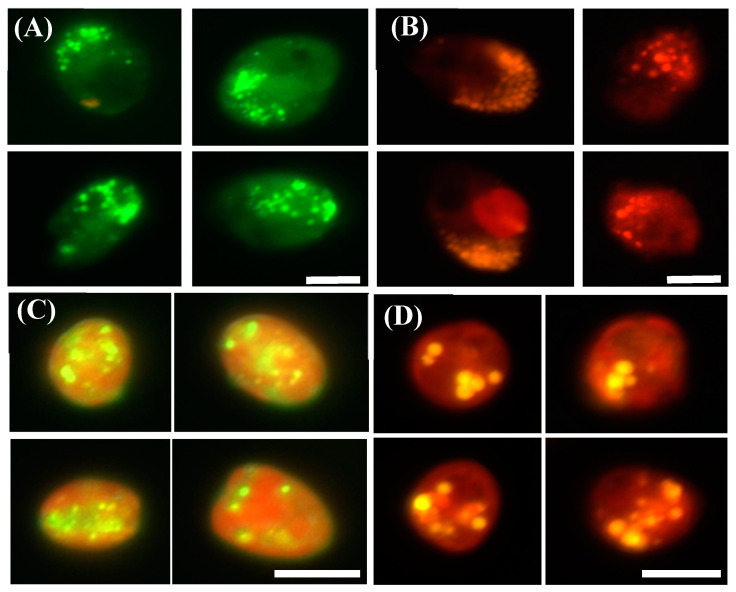
Lipid bodies were labeled with a lipophilic fluorescent dye under an epifluorescence microscope. (**A**) Heterotrophic *Oxyrrhis marina* labelled with BODIPY 505/515 was observed using an FITC (green emission) long-pass filter. (**B**) *O. marina* labelled with Nile Red was observed using a rhodamine (red emission) filter. (**C**) *Prorocentrum cordatum* labelled with BODIPY 505/515, observed using an FITC (green emission) long-pass filter. Orange-red fluorescence indicates chlorophyll autofluorescence. (**D**) *P. cordatum* labelled with Nile Red using a rhodamine (red emission) filter. The lipid bodies showed different colors (orange) with chlorophyll autofluorescence (red). Scale bar = 10 µm.

Recent research has identified BODIPY 505/515 as a superior marker to Nile Red for visualizing lipid content [26,34]. We compared the heterotrophic dinoflagellate *O. marina* and the mixotrophic dinoflagellate *P. cordatum* after staining with both Nile Red and BODIPY 505/515 and observed them under an epifluorescence microscope (Figure 5). In *O. marina,* regardless of whether Nile Red or BODIPY 505/515 was used, only lipid droplets were visible, owing to the absence of pigments (Figure 5A,B). In contrast, in *P. cordatum*, orange-red fluorescence was observed across the cell under both the FITC long-pass and rhodamine filters, indicating background interference from cellular pigments (Figure 5C,D).

**Figure 6:**
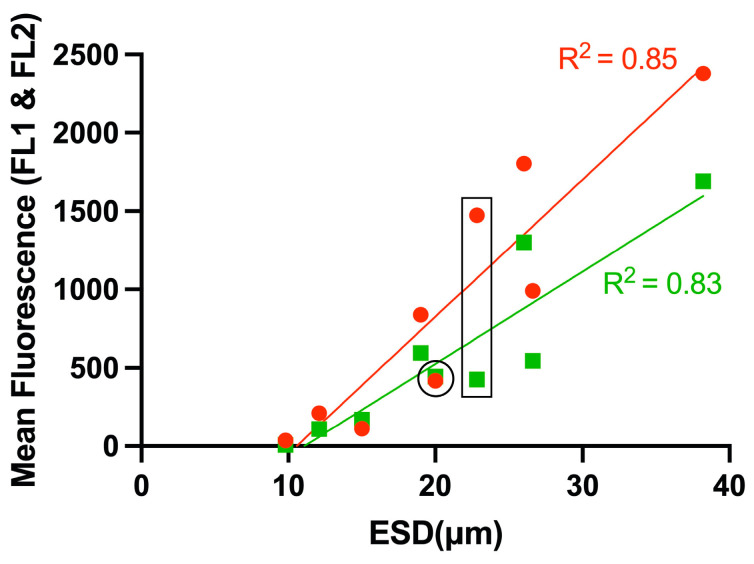
Comparison of lipid content of nine dinoflagellate species labelled with BODIPY 505/515 and Nile Red. Red and green indicate Nile Red and BODIPY 505/515 measurements, respectively. The slope of the linear correlation between the size and lipid content was steeper with Nile Red stain. Open circles indicate *Oxyrrhis marina*, which has no pigment (heterotrophic), and the elongated rectangle indicates *Scrippsiella acuminate.* R^2^ value is 0.85 and 0.83, respectively.

When we measured FL2 fluorescence in cells stained with Nile Red, *S. acuminata* exhibited significantly higher FL2 fluorescence than similarly sized species (Figure 6). *S. acuminata* has a thinner theca than other experimental species and displays very high chlorophyll autofluorescence when observed under an epifluorescence microscope. In contrast, the heterotrophic dinoflagellate *O. marina* showed relatively lower FL2 fluorescence than the similarly sized species, likely because it has no chlorophyll pigment (Table 2, Figure 6).

We also compared the mean fluorescence of experimental species and their equivalent spherical diameters (ESD) when stained with Nile Red and BODIPY 505/515 (Figure 6). The fluorescence intensity of Nile Red showed a linear correlation with ESD (R^2^ = 0.85), and the correlation coefficient was similar than that observed for BODIPY fluorescence (R^2^ = 0.83). While both stains exhibited a linear correlation between mean fluorescence and ESD, the Nile Red correlation exhibited a steeper gradient, indicating a potential overestimation of lipid content with this dye.

### 3.3. Estimating Lipid Content Using Relative Fluorescence

**Figure 7:**
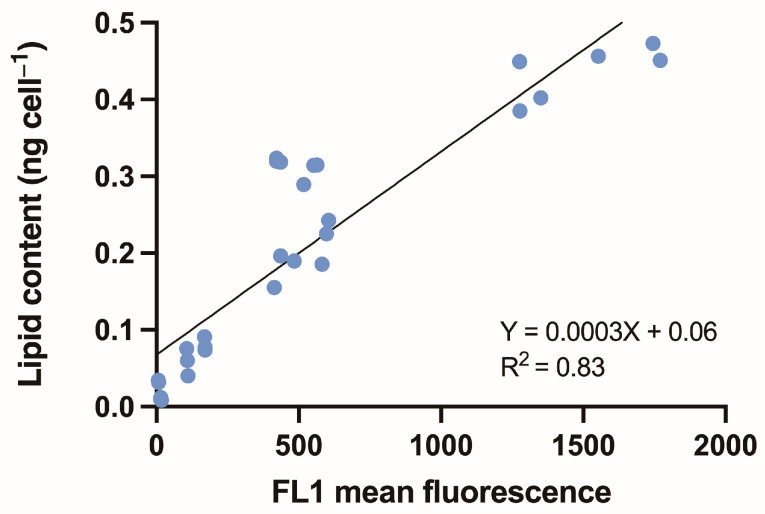
Correlation between BODIPY 505/515 fluorescence and lipid content estimated by the sulfo-phosphor-vanillin reaction in nine dinoflagellate species (three readings per species). The actual lipid content could be estimated multiplying FL1 fluorescence by 0.0003 and adding 0.06. R^2^ value is 0.83.

The formation of lipids is strongly correlated with algal growth, which is affected by environmental conditions such as light intensity and nutrient concentration [35,36,37]. Changes in light intensity can positively or negatively affect lipid production. Moreover, nitrogen starvation has often been reported to lead to an increase in lipid accumulation [38]. Therefore, it is crucial to monitor changes in lipid content during cultivation using rapid and simple methods to optimize the conditions for mass production of microalgae [5,19]. This study revealed a significant correlation between the relative lipid content estimated by flow cytometry using BODIPY 505/515, and the lipid content determined by the sulfo-phospho-vanillin (SPV) reaction in the experimental species (Table 2, Figure 7). The actual lipid content was estimated using the established flow cytometry conditions and BODIPY 505/515 staining by applying the slope value (multiplying FL1 fluorescence by 0.0003 and adding 0.06). Thus, the flow cytometry configurations established in this study offer an efficient and rapid flow cytometric technique for estimating lipid content in dinoflagellate species.

Previous studies have demonstrated a strong correlation between flow cytometric determination of lipid content and direct analytical methods, such as the Blight–Dyer extraction method or gas chromatography [16,39]. Our study revealed a strong correlation between flow cytometry and the SPV method for determining lipid content. Therefore, both flow cytometry and the SPV method are effective techniques that require only a small amount of biological material, enabling rapid quantification of dinoflagellate lipids without complex extraction steps.

### 3.4. Comparison of Relative Fluorescence with Size, Lipid Concentration and Carbon Content

Dinoflagellates play an important role as primary producers in marine food webs by converting atmospheric carbon dioxide into organic matter via photosynthesis [40,41]. A portion of this organic matter is stored within the cells in the form of lipids [42,43]. Dinoflagellate sterols, a component of their lipids, are often used as biomarkers to understand their productivity and ecological roles [44]. Estimating the carbon retention of individual plankton species as a primary producer in marine ecosystem is essential for assessing their productivity and its influence on carbon dynamics and the global carbon cycle [45]. In this study, we aimed to compare lipid content with carbon content to evaluate their applicability in productivity and ecological studies.

**Figure 8:**
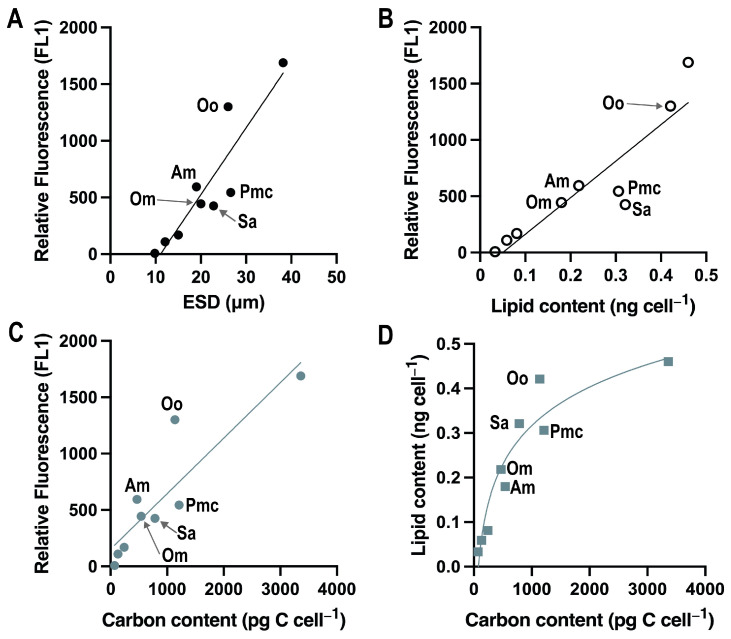
Correlations between BODIPY 505/515 fluorescence (FL1) and size (ESD; (**A**)), lipid content ([20]; (**B**)) and carbon content (**C**) of nine dinoflagellate species, and correlation between lipid content and carbon content (**D**). Oo = *O. ovata*; Am.= *A. minutum*; Pmc, *P. micans*; Sa, *S. acuminata*; Om, *O. marina*.

We compared the relative lipid content measured by flow cytometry with the carbon content calculated based on the cell size. When we compared the relative fluorescence (FL1) with size, lipid content, and carbon content, some species deviated from the general trend (Figure 8).

In a comparison between *A. minutum* and *O. marina*, which have similar sizes, the mixotrophic *A. minutum* exhibited a 20% higher lipid content than the heterotrophic *O. marina*. Similarly, when comparing *O. ovata* and *P. micans*, both capable of photosynthesis, the relative fluorescence of *O. ovata* was 2.4 times higher, and its lipid content was 40% greater than that of *P. micans* (Figure 8, Table 2). The relatively low lipid content of heterotrophic dinoflagellates (in the absence of prey) and high lipid content in benthic dinoflagellates suggest that the nutritional mode and habitat of dinoflagellates influence their cellular lipid content. The majority of dinoflagellate species used in this study had a spherical shape; therefore, the carbon content calculated based on cell volume and ESD showed a similar trend. Figure 8D illustrates a positive, non-linear correlation between carbon content (pg C cell^−1^) and lipid content (ng cell^−1^), with an R^2^ value of 0.90, signifying a strong relationship. The lipid content initially increased rapidly as the carbon content increased, but plateaued at higher carbon levels, suggesting a saturation effect.

## 4. Conclusions

This study demonstrates the effectiveness of flow cytometry with BODIPY 505/515 in estimating the lipid content of dinoflagellates. These findings demonstrate a strong correlation between the relative fluorescence, lipid content, and cell size, facilitating accurate lipid quantification. A straightforward equation was derived to estimate actual lipid content: 0.0003 × FL1 + 0.06. Furthermore, the relationship between the carbon content and lipid content revealed a saturation effect at higher carbon levels. These results underscore the value of flow cytometry as a rapid and efficient method for estimating lipid and carbon contents across diverse dinoflagellate species, offering a significant advantage over conventional extraction-based methods for large-scale studies.

## Figures and Tables

**Table 1:** List of experimental organisms, trophic modes, and sources.

Class	Species Name	Trophic Mode	Origin
Dinoflagellates	*Amphidinium carterae*	Mixotroph	USA
	*Kryptoperidinium triquetrum*	Mixotroph	Masan bay, Republic of Korea
	*Prorocentrum cordatum*	Mixotroph	Kunsan, Republic of Korea
	*Alexandrium minutum*	Mixotroph	CCMP 113
	*Oxyrrhis marina*	Heterotroph	Kunsan, Republic of Korea
	*Scrippsiella acuminata*	Mixotroph	Jeju, Republic of Korea
	*Ostreopsis ovata*	Autotroph	Jeju, Republic of Korea
	*Prorocentrum micans*	Mixotroph	Shiwha, Republic of Korea
	*Lingulaulax polyedra*	Mixotroph	USA

**Table 2:** Mean equivalent spherical diameter (ESD, μm), relative fluorescence (FL1, FL2) measured by flow cytometry (SSC, FL1 with BODIPY 505/515, FL2 with Nile Red), actual lipid content analyzed using the sulfo-phospho-vanillin (SPV) method [19] and carbon content of the experimental species.

Species Name	ESD (μm)	Relative Fluorescence (Average)			Lipid Conc. (ng Cell^−1^)	Carbon Contents (pg Cell^−1^)
		SSC	FL1 (BODIPY 505/515)	FL2 (Nile Red)	[19]	
*Amphidinium carterae*	9.8	229.4	6.8	36.8	0.033 ± 0.004	72.8
*Prorocentrum cordatum*	12.1	930.5	108.6	210.8	0.059 ± 0.018	131.8
*Krytoperidinium triquetrum*	15	641.6	169.7	111.0	0.081 ± 0.009	241.4
*Alexandrium minutum*	19	1185.8	594.4	838.4	0.218 ± 0.029	469.9
*Oxyrrhis marina*	20	1696.2	444.6	417.1	0.180 ± 0.022	542.9
*Scrippsiella acuminata*	22.8	1224.5	426.6	1474.8	0.321 ± 0.002	785.3
*Ostreopsis ovata*	26	2117.2	1301.4	1803.0	0.421 ± 0.033	1136.9
*Prorocentrum micans*	26.6	1447.1	544.7	991.2	0.306 ± 0.015	1212.4
*Lingulaulax polyedra*	38.2	4319.7	1689.7	2378.4	0.460 ± 0.012	3360.5

## Data Availability

The original contributions presented in this study are included in the article. Further inquiries can be directed to the corresponding authors.
